# Redefining the role of surgery in early small-cell lung cancer

**DOI:** 10.1007/s00423-022-02631-4

**Published:** 2022-08-04

**Authors:** Fabian Doerr, Sebastian Stange, Maximilian Michel, Georg Schlachtenberger, Hruy Menghesha, Thorsten Wahlers, Khosro Hekmat, Matthias B. Heldwein

**Affiliations:** 1 Department of Cardiothoracic Surgery, University Hospital of Cologne, Kerpener Straße 62, 50931 Cologne, Germany; 2 Department of Thoracic Surgery, Regiomed-Klinikum Coburg GmbH, Cologne, Germany; 3 Institute of Zoology, Faculty of Mathematics and Natural Sciences, University of Cologne, Cologne, Germany

**Keywords:** Small-cell lung cancer, Surgery, Meta-analysis, 5-year survival

## Abstract

**Purpose:**

Resection is guideline recommended in stage I small-cell lung cancer (SCLC) but not in stage II. In this stage, patients are treated with a non-surgical approach. The aim of this meta-analysis was to assess the role of surgery in both SCLC stages. Surgically treated patients were compared to non-surgical controls. Five-year survival rates were analysed.

**Methods:**

A systematic literature search was performed on December 01, 2021 in Medline, Embase and Cochrane Library. Studies published since 2004 on the effect of surgery in SCLC were considered and assessed using ROBINS-I. We preformed *I*^2^-tests, Q-statistics, DerSimonian-Laird tests and Egger-regression. The meta-analysis was conducted according to PRISMA.

**Results:**

Out of 6826 records, we identified seven original studies with a total of 15,170 patients that met our inclusion criteria. We found heterogeneity between these studies and ruled out any publication bias. Patient characteristics did not significantly differ between the two groups (*p*-value > 0.05). The 5-year survival rates in stage I were 47.4 ± 11.6% for the ‘surgery group’ and 21.7 ± 11.3% for the ‘non-surgery group’ (*p*-value = 0.0006). Our analysis of stage II SCLC revealed a significant survival benefit after surgery (40.2 ± 21.6% versus 21.2 ± 17.3%; *p*-value = 0.0474).

**Conclusion:**

Based on our data, the role of surgery in stage I and II SCLC is robust, since it improves the long-term survival in both stages significantly. Hence, feasibility of surgery as a priority treatment should always be evaluated not only in stage I SCLC but also in stage II, for which guideline recommendations might have to be reassessed.

## Introduction

Lung cancer is the leading cause of cancer death. Approximately two million new cases were diagnosed worldwide in 2018 [1]. Though small-cell lung cancer (SCLC) accounts for only 10–15% of all malignant lung tumours, it is the fifth leading cause of cancer death [2]. A rapid growth of the primary lesion and an early spreading to mediastinal lymph nodes or distant organs is typically found in this high-grade malignant disease [2].

Historically, SCLC was staged into ‘limited’ and ‘extensive’ cancer according to Veterans Administration Lung Study Group. This categorization has influenced treatment algorithms worldwide. However, recent studies show survival differences according to the extent of the primary tumor and the nodal involvement of the disease [3]. Consequently, the current UICC/IASLC 8th edition staging system defines stage I SCLC as tumours ≤ 4 cm without lymph node involvement (T1-T2aN0) and stage II as T2b-T3N0 or T1-T2N1 disease [4].

Curative treatment is based on a multimodal approach combining chemotherapy, radiation and in selected cases surgery [5]. To date, the role of surgery is still under debate as there are no recent randomized controlled trials (RCTs) of surgery in SCLC [6]. Current recommendations are based on large dataset analysis which suggests a survival benefit after surgery. Consequently, a surgical approach is recommended only for stage I (T1-2aN0) disease by NCCN and ACCP guidelines [5, 7].

The aim of this study was to evaluate whether surgery would benefit not only in stage I but also in stage II disease. We conducted a meta-analysis and compared 5-year survival rates after surgical treatment in stage I and stage II SCLC with long-term survival of patients who underwent no surgical treatment.

## Material and methods

### Study inclusion and exclusion criteria

**Fig. 1:**
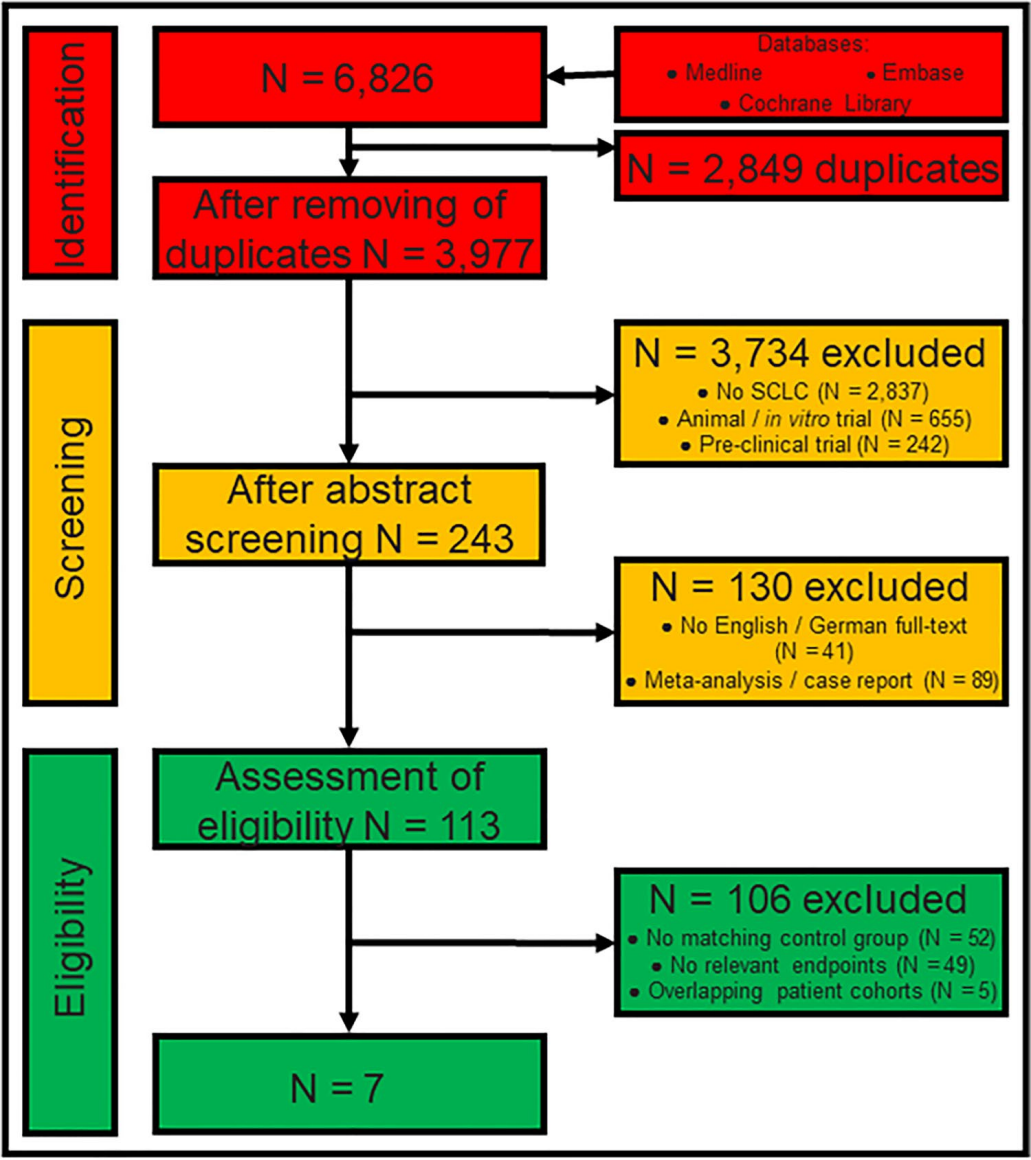
Flowchart of literature research. ‘Identification’ (red) shows how we selected studies after a literature research in three databases. We then ‘screened’ (orange) relevant articles which are finally ‘eligible’ (green) for inclusion into the meta-analysis. Coloured boxes in the middle display number of articles at each step of assessment. Coloured boxes in the right display the number of excluded articles and the reason of exclusion. SCLC small-cell lung cancer

We performed a systematic review in accordance with the Preferred Reporting Items for Systematic Reviews and Meta-Analyses (PRISMA) guidelines [8]. Databases were queried for studies on the effect of surgery in stage I and stage II SCLC published since 2004. With the availability of modern staging tools such as computed tomography (CT-scan), staging of lung cancer has changed over the past decades [9]. Therefore, we restricted inclusion to studies which recruited patients within the last 35 years to avoid bias due to inaccurate staging. We further employed inclusion criteria listed in Fig. 1. Briefly, studies involving (1) stage III or extensive (M1) stage SCLC, (2) lack of a control group, (3) animal trials, (4) in vitro trials, (5) trials with non-clinical end-points, (6) case reports, (7) editorials and (8) comments or guidelines were excluded.

We defined the ‘non-surgery group’ as radio-/chemotherapy treatment only. All patients who underwent surgical treatment alone or in combination with neoadjuvant or adjuvant therapy were defined as ‘surgery group’. The definitions of clinical end-points were taken from the primary publications.

### Search strategy

Two authors (FD and SS) performed an independent literature search on December 01, 2021 in the Medline, Embase and the Cochrane Library databases using the following predefined list of keywords: (((small cell lung cancer) OR (small cell lung carcinoma) OR (oat cell lung cancer)) AND (limited) AND ((resection) OR (surgery) OR (surgical) OR (chemotherapy) OR (radiation therapy) OR (radiotherapy) OR (radiochemotherapy))). We restricted the languages to English and German. A reference management software (Endnote, Version X9.2, Clarivate Analytics, Spring Garden, Philadelphia, USA) was used to organize all relevant articles. An initial selection was performed by reviewing all titles and abstracts. We recovered full text and further screened the reference lists of these papers to identify further publications fulfilling the above criteria. In cases where we found several publications per patient collective, we selected the study with the most complete dataset. With this measure, we aim to guarantee that patients are included only once despite compiling cohorts from similar data sources. Figure 1 provides a detailed flowchart of the search strategy.

### Data extraction and quality assessment

All relevant data of interest were extracted from the original studies. The first or the senior author of an original study was contacted in case of missing information. Study quality and risk of bias were assessed by two independent investigators (FD and SS) using ROBINS-I [10].

To avoid a staging bias, we evaluated all studies for identical staging conditions in the treatment groups. TNM classification was identical in both groups, and either pTNM or cTNM was analysed. A comparison of the more accurate pTNM in the surgery group with cTNM in the non-surgery group was avoided.

Not all original studies performed pair-matching in regard to the patients’ fitness. Therefore, we evaluated the performance score to assess the fitness of patients in both groups whenever available. Since such scores were not reported regularly, patients that underwent surgery might have had a better health status at the time of intervention compared to the non-surgical counterparts creating bias in selected cases.

### Statistical analysis

Statistical analysis was performed using the StatsDirect software package (Version 3.2.10, StatsDirect Ltd, Birkenhead, Merseyside, UK). Throughout our statistical analysis, a *p*-value < 0.05 was considered significant. For each individual study, we analysed either the raw incidence data of the clinical end-point or the estimated effects expressed as the odds ratio (OR). The 95% confidence interval (95%-CI) of each data set was calculated and expressed as a Forest plot. Q-statistics (*p*-value < 0.05) and *I*^2^-tests (*I*^2^ > 50%) were performed to evaluate heterogeneity between included studies [11]. When we found heterogeneity, we implemented the DerSimonian and Laird random-effects model [12]. In absence of heterogeneity, we used the Mantel–Haenszel fixed-effects model. The pooled treatment effect estimate was calculated as a weighted average of the treatment effects so that an OR > 1 favoured the surgery group over the control group. We assessed publication bias with Egger’s weighted regression statistic, with a *p*-value < 0.05 indicating significant publication bias among included studies. To quantify the impact of the surgical treatment, we calculated the absolute risk reduction (ARR), relative risk reduction (RRR) and number needed to treat (NNT). Finally, we collected the patients’ mean age from all studies and calculated a mean as well as standard deviation. The gender distribution represents weighted sums of the original data.

## Results

### Literature search

**Fig. 2:**
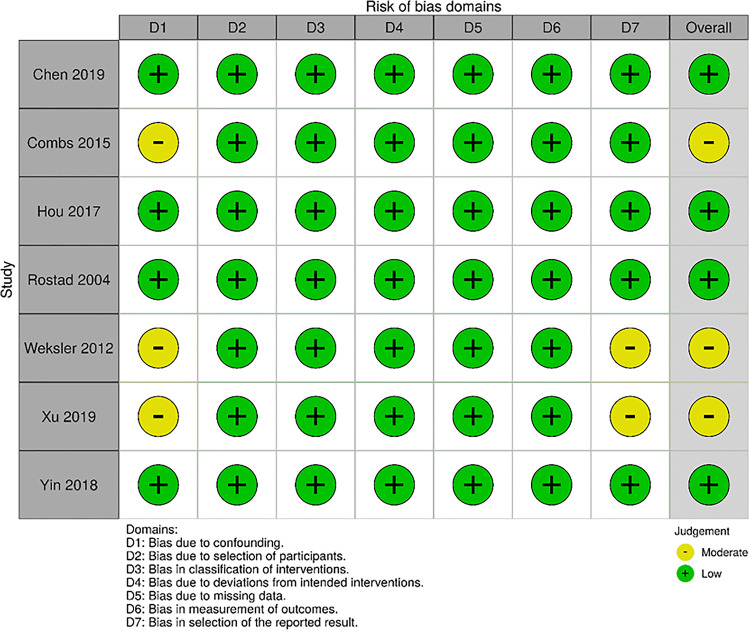
Overall risk of bias according to ROBINS-I

From an original set of 6826 papers found in the systematic literature research, seven studies were included in the meta-analysis (Fig. 1). According to ROBINS-I, the overall risk of bias in the studies included was low or moderate (Fig. 2). Publication dates ranged between 2004 and 2019 (Table 1). The longest period of patient recruitment was 20 years (1988–2007) in the study by Weksler et al. [13]. One of the most recent publications with the shortest recruitment period of 6 years was Yin et al. (2010–2015) [14]. All studies are retrospective. Four studies are based on national data registries and three studies compile single-centre data, of which the largest is that of Hou et al. with 208 patients published in 2017 [15]. Two original studies (Chen et al. and Yin et al.) used a pair-match analysis [14, 16] (Table 1).

**Table 1:** Overview of all original studies

Author	Year	Period	Origin	Comment	5-y surv. (%)		Patients (N)			Complete resection (%)
					Surg	NS	All	Surg	NS	
Chen	2019	2000–2016	SC	St. I, PM	62.3	40.1	58	30	28	95
Combs	2015	1998–2006	NCDB	St. I	47.0	18.0	4893	1009	3884	87
Combs	2015	1998–2006	NCDB	St. II	19.0	16.0	4787	235	4552	
Hou	2017	2005–2010	SC	St. I + II	50.9	44.3	208	102	106	91
Rostad	2004	1993–1999	CR	St. I	44.9	11.3	96	38	58	89
Weksler	2012	1988–2007	SEER	St. I + II	26.9	7.0	3566	895	2671	NR
Xu	2019	2010–2015	SEER	St. IA	43.9	19.2	547	163	384	NR
Xu	2019	2010–2015	SEER	St. IB	41.7	23.5	265	80	185	
Xu	2019	2010–2015	SEER	St. IIA	75.0	0.0	113	11	102	
Xu	2019	2010–2015	SEER	St. IIB	26.8	16.0	599	83	516	
Yin	2018	2010–2015	SC	St. II, PM	35.9	25.3	38	19	19	94
*Jin*	*2018*	*2004*–*2013*	*SEER*	*St. I* + *II*	*42.7*	*25.9*	*1186*	*154*	*1032*	*NR*
*Lin*	*2020*	*2004*–*2014*	*SEER*	*St. IA*	*50.0*	*24.7*	*686*	*337*	*349*	*NR*
*Schreiber*	*2010*	*1988*–*2002*	*SEER*	*St. I*	*52.6*	*13.7*	*2226*	*231*	*1995*	*NR*
*Varlotto*	*2011*	*1988*–*2005*	*SEER*	*St. I*	*47.4*	*17.2*	*1053*	*361*	*692*	*NR*
*Yang*	*2018*	*2003*–*2011*	*NCDB*	*St. I*	*48.1*	*28.3*	*2301*	*681*	*1620*	*93*

Summary of each original study including year of publication, period of patient recruitment, data origin, a comment on details of each original study including stage analysed, 5-year survival rates in %, and number of patients in each treatment group. Studies in the lower part of the table, displayed in italic were excluded from this meta-analysis due to overlapping patient cohorts from similar data sources

*5-y surv.* 5-year survival, *CR* Cancer registry (Norway), *NCDB* National Cancer Database, *NR* not reported, *NS* Non-surgery group, *PM* Pair-match analysis, *SC* Single centre, *SEER* Surveillance, Epidemiology, and End Results database, *St.* stage analysed, *Surg.* ‘Surgery group ‘

Despite meeting the inclusion criteria, we excluded five studies due to overlapping patient cohorts from similar data sources (SEER and NCDB). All data of the five excluded studies are displayed in the lower section of Table 1.

### Patient details

We included a total of 15,170 patients with stage I and II SCLC in this meta-analysis. Of these, 2665 patients are in the surgery group and 12,505 in the non-surgery group. Patients’ mean age was 66.3 ± 1.4 years, and 49.0 ± 5.4% of all patients were male. Patient characteristics did not significantly (*p*-value > 0.05) differ between the surgery group and non-surgery group (Tables 2 and 3). The stage I analysis is based on subgroup of 8596 patients (surgery: *n* = 2,032; non-surgery: *n* = 6,564). The stage II analysis is based on subgroup of 6574 patients (surgery: *n* = 633; non-surgery: *n* = 5941).

**Table 2:** Baseline characteristics

Number patients	Mean age (years)	*p*-value	Male (%)	*p*-value
All patients (15,170)	66.3 ± 1.4		49.0 ± 5.4	
Surg. group (2665)	67.0 ± 1.1	0.408	51.4 ± 7.9	0.371
Non-surg. group (12,505)	65.5 ± 1.9		47.7 ± 3.0	

Summary of patients’ baseline characteristics including the number of patients in each group, mean age in years and gender distribution expressed as % male

*Non-surg.* non-surgery group, *Surg.* surgery group

**Table 3:** N0-/N1-distribution of patients

Author	Chen		Combs		Hou		Rostad		Weksler		Xu		Yin	
Stage	I		I + II		I + II		I		I + II		I + II		II	
Group	S	NS	S	NS	S	NS	S	NS	S	NS	S	NS	S	NS
N0 (%)	100	100	73	69	52	47	100	100	84	83	81	77	13	14
N1 (%)	0	0	27	31	48	53	0	0	16	17	19	23	87	86

*NS* non-surgery group, *S* surgery group

### Five-year survival analysis in stage I

**Fig. 3:**
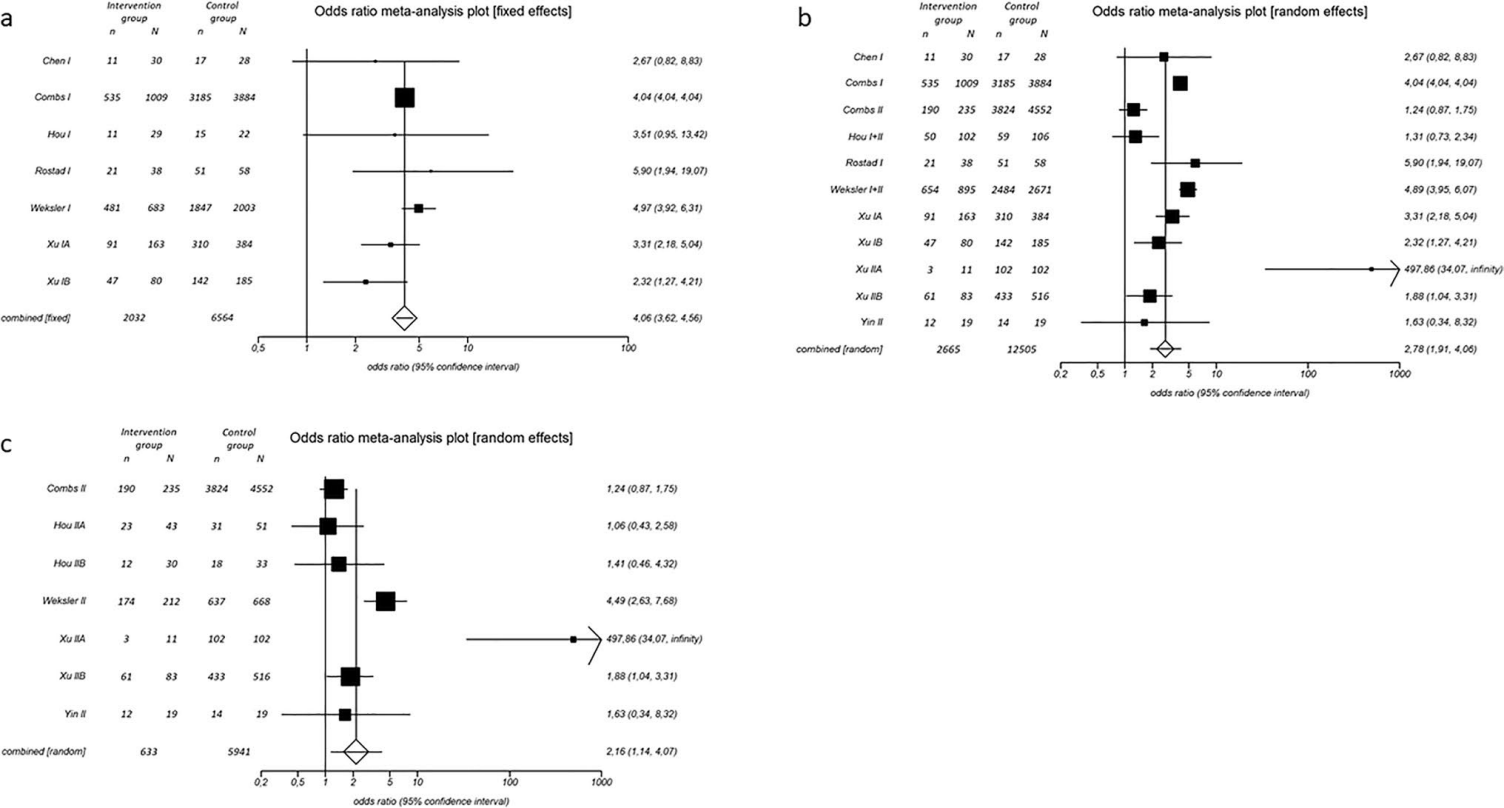
Summary meta-analysis plot in stage I (**a**), in stage I and II combined (**b**), and in stage II isolated (**c**). The figure displays the results of the meta-analysis in stage I (**a**), combined stage I and II (**b**), and separate stage II (**c**). Names on the left stand for first author of original study. Studies were mentioned multiple times in cases where different SCLC stages were included in one analysis. Intervention group: surgery. Control group: non-surgery. n: number in group with outcome. N: Total number in group. Odds ratio > 1 provides evidence for superiority of surgery. Size of squares indicates the sample size. Numbers on the right display odds ratio and 95%-confidence interval for each study

The Q-statistic for the 5-year survival endpoint was not significant (*p*-value = 0.1768) and the *I*^2^-test suggested 32.9% inconsistency (95%-CI: 0–70.9%), providing evidence for the absence of heterogeneity. We therefore implemented the Mantel–Haenszel fixed-effects model. The pooled odds ratio was 4.1 (95%-CI: 3.6–4.6) and the Chi^2^ was 627.0 (*p*-value < 0.0001). This result suggests that the surgery group showed significant improvement in the 5-year survival endpoint compared to control patients (Fig. 3a). Egger’s weighted regression statistic showed no significant publication bias (*p*-value = 0.4244). Surgical intervention improved 5-year survival significantly (*p*-value = 0.0006). The 5-year survival rates were 47.4 ± 11.6% in the surgery group versus 21.7 ± 11.3% in the non-surgery group (Table 4; Fig. 3a). The ARR was 25.7 ± 5.5%, and the RRR was 33.2 ± 7.7%. For one patient to reach a 5-year survival in stage I SCLC, five patients need to be resected (NNT: 4.1) (Table 4).

**Table 4:** Results of the meta-analysis

Stage	Group	Number of patients	5-year survival		ARR (%)	RRR (%)	NNT
			%	*p*-value			
I	Surgery	2032	47.4 ± 11.6	0.0006	25.7 ± 5.5	33.2 ± 7.7	4.1
	Non-Surgery	6564	21.7 ± 11.3				
I + II	Surgery	2665	43.1 ± 16.2	0.0008	23.1 ± 19.7	27.6 ± 19.5	8.6
	Non-Surgery	12,505	20.1 ± 13.1				
II	Surgery	633	40.2 ± 21.6	0.0474	18.9 ± 25.0	22.2 ± 24.1	12.0
	Non-Surgery	5941	21.2 ± 17.3				

Summary of meta-analysis including 5-year survival rates in % for stage I, combined stage I and II, and separate stage II

*ARR* absolute risk reduction in %, *NNT* number needed to treat, *RRR* relative risk reduction in %

### Five-year survival analysis in stage I and II combined

Since several groups consider surgical treatment for patients in stage II SCLC, it is plausible to include this sub-population in an analysis. We therefore analysed 5-year survival specifically in stage I and II. The Q-statistic for the 5-year survival endpoint was significant (*p*-value < 0.0001), and the *I*^2^-test suggested 87.9% inconsistency (95%-CI: 80.3–91.6%), providing evidence for significant heterogeneity between the included studies. We therefore implemented the DerSimonian and Laird random-effects model. The pooled odds ratio was 2.8 (95%-CI: 1.9–4.1), and the Chi^2^ was 28.1 (*p*-value < 0.0001). This suggests that patients in stage I and II SCLC had significantly improved 5-year survival after surgery (Fig. 3b). Egger’s weighted regression statistic showed no significant publication bias (*p*-value = 0.4284). Surgical intervention improved 5-year survival in both stages significantly (*p*-value = 0.0008). The 5-year survival rates were 43.1 ± 16.2% in the surgery group versus 20.1 ± 13.1% in the non-surgery group (Table 4; Fig. 3b). The ARR was 23.1 ± 19.7%, and the RRR was 27.6 ± 19.5%. For one patient to reach a 5-year survival in stage I or II SCLC, nine patients need to be surgically treated (NNT: 8.6) (Table 4).

### Five-year survival analysis in isolated stage II

In order to show that the survival advantage in the combined analysis of both stages is not exclusively due to the good results of stage I, we analysed stage II separately. Here, the Q-statistic for the 5-year survival endpoint was significant (*p*-value < 0.0001), and the *I*^2^-test suggested 81.5% inconsistency (95%-CI: 57.4–89.3%), again showing significant heterogeneity between included studies. We applied the DerSimonian and Laird random-effects model as in the other analysis. The pooled odds ratio was 2.2 (95%-CI: 1.1–4.1), and the Chi^2^ was 5.6 (*p*-value < 0.0177). These results suggest a significant survival benefit for patients in stage II after surgery (Fig. 3c). Egger’s weighted regression statistic showed no significant publication bias (*p*-value = 0.3226). Consequently, surgical intervention improved 5-year survival in isolated stage II SCLC (*p*-value = 0.0474). Five-year survival was 40.2 ± 21.6% in the surgery group versus 21.2 ± 17.3% in the non-surgery group (Table 4; Fig. 3c). The ARR was 18.9 ± 25.0%, and the RRR was 22.2 ± 24.1%. For one patient to reach a 5-year survival in stage II SCLC, 12 patients need to be surgically treated (NNT: 12.0) (Table 4).

## Discussion

### Two historic RCTs made up the case against surgery in SCLC

The two RCTs that became landmark studies in the decision against surgery as first treatment option in SCLC were carried out by Fox et al. [17] and Lad et al. [18]. The study of Fox et al. [17] was published in 1973 and is often referred to as the turning point in SCLC treatment. In this Medical Research Council (UK) trial, patients were randomized to receive either surgery or radiotherapy. At that particular time, modern imaging techniques namely computed tomography (CT-scan), PET-scan or diagnostic tools such as EBUS were not available. Most patients in both treatment arms likely had undetected advanced cancer and would not be suitable for surgery today. Furthermore, in only 48% of all patients that were randomized to surgery, a R0 resection was achieved by performing a pneumonectomy. Among the remaining patients, 34% received an explorative thoracotomy without any resection (R2-situation). Lastly, 18% of patients in the surgical arm did not have surgery at all because they either deteriorated and were found unfit for surgery or decided not to have any resection [17]. Consequently, patients in the non-surgery group fared better, and the standard of care was changed from surgery to radiotherapy [13, 17, 19].

Two decades later, Lad et al. randomized SCLC patients to surgery or non-surgery following chemotherapy and radiotherapy [18]. The median survival was 15.4 months for the surgery group and 18.6 months for the non-surgery group. The authors postulated that surgery had no survival benefit. However, 11% of patients that were randomized to the surgical arm refused the operation and consequently were not resected at all. Among the patients that underwent an operation after randomization, 17% were found to be unresectable after explorative thoracotomy (R2-situation) and 6% were incompletely resected (R1). In 77% of the patients that underwent surgery, a complete resection was achieved [18]. Furthermore, only patients with regional lymph node involvement were considered for surgery in this study, and patients in early disease stages, who would be ideal candidates for resection today, were excluded [13, 18, 19]. Both randomized trials had a strong impact on the treatment protocol of SCLC and led to a negligence of surgery for several decades.

### Recent data argues for surgery in SCLC — what has changed?

In our meta-analysis, we were able to demonstrate an improved outcome following surgery compared to non-surgical therapy. This is in contrast to the above-mentioned RCTs, and this could be due to changing surgical techniques and non-surgical treatment options as well as improved and more accurate staging over the past decades. In some recent studies, surgery was performed as muscle sparing thoracotomy or as VATS with user friendly tissue staplers, possibly improving outcome. A time bias should therefore be considered as a factor generally influencing the prognosis of SCLC [20]. It is of note that studies published before the year 2000 seem to show a reduced impact of surgery on survival benefit compared to later studies [21–23]. Both Takenaka et al. and Zhang et al. postulate that long-term outcomes in any SCLC stage have gradually improved beginning in the 2000s [20, 24]. Nonetheless, in the latest review article on SCLC published a few months ago, Rudin et al. see no role for surgery beyond stage I [25].

### Does this meta-analysis justify redefining the role of surgery in stage I and II SCLC?

We provide a large meta-analysis of more than 15,000 patients. All studies that were sourced for this meta-analysis showed good data quality. We were able to rule out publication bias among the included studies, and patient characteristics did not differ significantly between groups. We revealed significantly improved survival after surgery in stage I SCLC with 5-year survival rates of 47.4% after resection and 21.7% without surgery. In the light of a 19% survival benefit in stage II SCLC, surgical therapy becomes a stronghold in stage I and II against current guideline recommendations, according to which surgery is only recommended in stage I [5, 7]. Some of the studies included in this meta-analysis deliver remarkable results. The 5-year survival benefit of stage I SCLC patients varied between 21.8% (Weksler et al.) [13] and 33.6% (Rostad et al.) [23]. The study by Combs et al. with the largest cohort of 4,893 stage I patients showed a 5-year survival benefit of 29.0% in favour for the surgically treated patients [26]. In the two studies that analysed a matched patient collective, the 5-year survival benefit was 22.2% in stage I [16] and 10.6% in stage II SCLC [14]. It is relevant to note that Xu et al. examined the sub-stages separately in relation to 5-year survival. The authors noted a significantly improved survival in all sub-stages, even with no surviving patient in stage IIA after non-surgical treatment [27].

It is not to be expected that the findings of our meta-analysis would significantly change as a result of including the five studies that were excluded due to overlapping patient cohorts from similar data sources, as these studies support our data. The SEER database was sourced for patients by Varlotto et al., Schreiber el al. and Lin et al. for stage I patients, and by Jin et al. (stage I and II). The 5-year survival benefit after resection ranged between 25 and 40% in stage I patients [28–30] and reached 17% in stage I and II [31]. Similar to other authors, the survival analysis by Yang et al. is based on patient data from the NCDB database and reached a 20% survival benefit after 5 years in stage I [32]. Furthermore, Zhu et al. published a single-centre study in 2013 and reported a significant 5-year survival benefit for surgically treated patients in stage I and II compared to patients treated with chemoradiotherapy (57.0% versus 31.4%; *p*-value: 0.004) [33]. This study could not be included into our meta-analysis since some relevant data were not reported by the authors.

We believe that the relevance of surgery to treat SCLC patients is currently underestimated. Our meta-analysis might help to move surgery into the centre of early-stage SCLC treatment since its impact on long-term survival is robust.

### Improving surgery: how extensive does an SCLC patient have to be staged?

Due to early distant metastasis, a precise staging at the time of treatment initiation is of high importance in SCLC patients [34, 35]. According to NCCN and ACCP guidelines, a spiral computed tomography (CT) scan of the chest and abdomen with intravenous contrast and magnetic resonance imaging (MRI) or a CT-scan of the brain are recommended (Grade 1B) [5, 7]. The staging workup should include PET imaging for patients in a clinically limited stage (Grade 2C). Invasive mediastinal staging (tissue examination of mediastinal nodes) is recommended in patients who are being considered for surgical resection with curative intent (Grade 1B) [5, 7], since aggressive mediastinal staging and pathologic nodal evaluation based on EBUS fine needle aspiration or mediastinoscopy helps in identifying potential surgical patients and occult nodal disease [34, 35].

### Improving surgery: what is the best technique?

Since we show mounting evidence for the benefit of surgery in the treatment of SCLC, it becomes relevant to identify the most beneficial surgical procedure. Combs et al. report on the benefit of performing a lobectomy for 5-year survival rates. The authors compare survival after lobectomies with survival after sublobar resection and pneumonectomy (5-year survival: 40%, 21% and 22%, respectively) [26]. The data of Weksler et al. similarly indicate that wedge resection results in significantly worsened median survival compared to lobectomy or pneumonectomy (39 months versus 28 months, *p*-value < 0.001) [13]. According to Schreiber et al., the median survival time was longest after lobectomy, followed by sublobar resection, pneumonectomy and lack of surgery (40 months, 23 months, 20 months and 13 months, respectively) [30]. Lastly, Lüchtenborg et al. show reduced outcomes for patients after pneumonectomy compared to lobectomy or bilobectomy (adjusted HR 1.53; 95%-CI: 1.05–2.25) [19]. In attempt to avoid a pneumonectomy, it is not uncommon to perform a sleeve resection in SCLC due to centrally growing tumours [36]. Since it is important to assess the need of postoperative radiation of the mediastinum, a systematic lymph-node dissection should always be performed besides a lobectomy [37, 38].

## Conclusion

We are the first group demonstrating a significant 5-year survival benefit in a meta-analysis on such a large scale of stage I and stage II SCLC patients. Based on the presented survival data, we suggest that guidelines should consider the role of surgery as priority treatment option in early-stage SCLC disease not only in stage I but also in stage II.
